# Is targeted fortification of human breast milk an optimal nutrition strategy for preterm infants? An interventional study

**DOI:** 10.1186/s12967-016-0957-y

**Published:** 2016-07-01

**Authors:** Laura Morlacchi, Domenica Mallardi, Maria Lorella Giannì, Paola Roggero, Orsola Amato, Pasqua Piemontese, Dario Consonni, Fabio Mosca

**Affiliations:** Neonatal Intensive Care Unit, Department of Clinical Science and Community Health, Fondazione IRCCS Ca’ Granda Ospedale Maggiore Policlinico, University of Milan, Via Commenda 12, 20122 Milan, Italy; Epidemiology Unit, Fondazione IRCCS Ca’ Granda Ospedale Maggiore Policlinico, Via Commenda 12, 20122 Milan, Italy

**Keywords:** Human milk target fortification, Preterm infants, Growth rate, Osmolality

## Abstract

**Background:**

Fortifying human milk contributes to the prevention of postnatal growth failure in preterm infants. Because of the natural variability of human milk, targeted fortification of human milk has been advocated. However, data regarding the efficacy and safety of prolonged targeted fortification are scarce. We aimed to assess the safety of targeted fortification of human milk in preterm infants compared with standard fortification, as well as the effects on infant growth.

**Methods:**

We conducted an interventional study during hospital stay in healthy very low birth weight preterm infants who were exclusively fed human milk. Pools of human milk collected for 24 h were analysed using mid-infrared transmission spectroscopy. Targeted fortification of human milk was performed by adding macronutrients to native human milk to obtain optimal ratios of fat (4.4 g), carbohydrates (8.8 g), and protein (3 g) per 100 ml. The intervention period lasted 4–7 weeks. Weekly weight and daily growth rates were compared with those of a standardized fortification group of very low birth weight preterm infants who received standard fortified human milk (n = 10). The osmolality as well as the metabolic and gastrointestinal tolerance were monitored. Intergroup differences were evaluated using the Mann–Whitney U-test.

**Results:**

A total of 10 preterm infants (birth weight 1223 ± 195 g; gestational age 29.1 ± 1.03 weeks) were enrolled and 118 samples of pooled milk were analysed. On average, 1.4 ± 0.1 g of protein, 2.3 ± 0.5 g of carbohydrate, and 0.3 ± 0.1 g of fat per 100 ml were added to the milk. Osmolality values after target fortification were within recommended limits (376 ± 66 mOsml/kg). Weekly weight gain (205.5 g; 95 % CI 177–233 vs 155 g; 95 % CI 132–178; p = 0.025) and daily growth rates (15.7 g/kg/day; 95 % CI 14.5–16.9 vs 12.3 g/kg/day; 95 % CI 10.7–13.9; p = 0.005) were higher in infants receiving target fortification than in infants receiving standardized fortification. The infants receiving targeted fortified milk consumed similar volumes as infants in the standardized fortification group (148 ± 4.5 vs 146 ± 4 ml/kg/day). No signs of either gastrointestinal or metabolic intolerance were observed.

**Conclusions:**

Target fortification appears to promote growth in very low birth weight preterm infants without any detrimental effects.

*Trial registration* NCT02716337

## Background

The American Academy of Paediatrics recommends that the postnatal nutritional management of very low birth weight (VLBW, birth weight <1500 g) infants should meet the intrauterine rates of foetal growth, i.e., 15 g/kg/day [1]. However, despite several efforts in recent years regarding nutritional support of preterm infants, postnatal growth restriction is still common in neonatal intensive care units [2]. Increasing evidence indicates that adequate postnatal growth is positively associated with better neurodevelopmental outcomes [3]. Therefore, the prevention of postnatal growth restriction in preterm infants during hospitalization is of the utmost importance.

Breast milk is acknowledged as ideal for the nutritional support of preterm infants because of its several health benefits on immunological, gastrointestinal and neurodevelopmental functions

The implementation of human milk feeding has been associated with improved feeding tolerance, reduced rates of necrotizing enterocolitis, sepsis and retinopathy of prematurity, in addition to promotion of neurocognitive development [4].

However, although the protein and energy content of breast milk from mothers who delivered prematurely is higher than that from mothers of term infants, preterm milk does not meet the nutritional requirements of preterm infants [5]. Moreover, inter- and intra variability of breast milk composition is elevated [6, 7]. To optimize the nutritional composition of maternal milk and provide preterm infants with adequate intake of macronutrients, the addition of commercially available fortifiers, either as a single component or multicomponent, has been advocated. Improved growth rates and nutritional parameters have been reported in preterm infants exclusively fed fortified breast milk compared with preterm infants exclusively fed unfortified breast milk [5]. However, fortification of breast milk may cause adverse effects, including metabolic acidosis [8]. With regard to osmolality, the combination of multicomponent and single component fortifiers can cause an increase over the recommended upper limits, thus increasing the risk of gastrointestinal intolerance [9, 10]. Currently, three strategies of breast milk fortification are available [11]. Standard fortification is the most commonly used practice of breast milk supplementation, which is based on the addition of a fixed dose of product and assuming a standard composition of breast milk. A second approach, called adjustable fortification, modulates protein supplementation on the basis of blood urea nitrogen levels [12]. The target fortification strategy is based on the regular analysis of breast milk composition, thus allowing for the provision of adequate individual targeted macronutrients to each infant. In recent years, reports have suggested the benefits of this customized approach on decreasing the variability of maternal milk’s macronutrients [13] and improving growth in preterm infants [14, 15]. However, studies exploring the efficacy and safety of prolonged targeted fortification compared with standard fortification in VLBW preterm infants are still scarce.

The aim of this study was to assess the growth benefits of targeted breast milk fortification during the hospital stay compared with standard fortification in VLBW preterm infants. The hypothesis was that VLBW preterm infants receiving targeted fortification would show better growth at the time of discharge without adverse effects concerning safety and metabolic tolerance.

## Methods

### Study design and subjects

A prospective interventional study was conducted in the NICU of Mangiagalli Clinic in Milan, Italy. Qualifying infants born between October 2014 and March 2015 were enrolled in the intervention group. Informed written consent was obtained from the parents of the participants before enrolment.

The inclusion criteria were as follows: gestational age <32 weeks, birth weight <1500 g and ≥10th percentile according to Fenton**’**s growth chart [16] and exclusively consuming breast milk during the entire hospital stay. The exclusion criteria were the presence of either congenital or chromosomal abnormalities or conditions that interfere with growth such as chronic lung disease (as defined based on the classification of Jobe and Bancalari [17]), neurological disorders, metabolic, cardiac and gastrointestinal diseases and/or sepsis, which was defined as a positive blood culture.

**Fig. 1:**
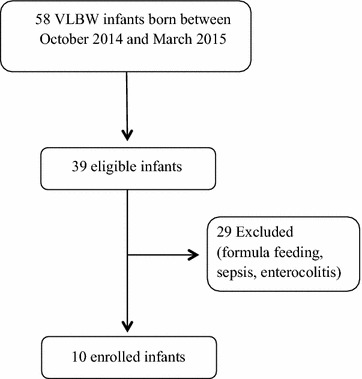
Flow chart of the study

A flow chart summarizing the study design is shown in Fig. 1.

Infants admitted to the same institution during the previous 6 months (January to June 2014) and who fulfilled the inclusion criteria of the study comprised the standardized fortification group.

### Nutritional practices

The nutritional management at our institution did not undergo major changes from January 2014 up to the intervention period except for the approach to breast milk fortification. According to the internal nutritional procedure of our institution, parenteral nutrition was started immediately after birth in infants in the standardized fortification and the intervention groups and enteral nutrition was administered as soon as possible. Nutritional management was similar in the two groups up to the initiation of breast milk fortification. Parenteral nutrition and minimal enteral feeding was started on the first day of life in all infants. The parenteral solutions were prepared by the hospital pharmacy according to the prescription. The solutions contained a minimum of 57 kcal/kg/day with 2.5 g/kg of protein on the first day of life and up to 90–100 kcal/kg/day and 4 g/kg/day of protein within the first week [18].

In both groups, fortification of breast milk was started when the enteral intake reached 90 ml/kg/day. The volume of enteral feeding was increased based on the infants’ cardio-respiratory stability and gastrointestinal tolerance.

### Milk fortification

Infants in the intervention group received individualized targeted fortification. To enhance feeding tolerance, targeted fortification was gradually introduced over a 3-day period. On day 4, the full amount of the targeted fortification for each macronutrient was prescribed. The intervention period lasted until the infant was discharged.

Mothers of enrolled infants collected expressed milk in sterile bottles. Every monday and thursday at 7 a.m., nurses mixed a bottle containing the milk collected during the previous 24 h using a vortex shaker. Approximately 2 ml of breast milk from each bottle was aliquoted for the macronutrient analysis. Prior to composition analysis, samples of native breast milk were frozen [19]. After 24 h, the thawed milk was homogenized using an ultrasonic homogenizer (Sonicator^®^, Uppsala, Sweden). Analyses were performed using a human milk analyser (Miris AB^®^, Uppsala, Sweden) based on mid-infrared transmission spectroscopy. The instrument was calibrated for breast milk measurements using the Kjeldahl method for proteins, HPLC for lactose and the Rose-Gottlieb method for fat [19].

The breast milk analyses were conducted twice a week because this measurement frequency has been reported to adequately compensate the daily nutritional variability of breast milk and to ensure a correct mean target of nutrient intake [20]. To comply with the guidelines from the European Society for Paediatric gastroenterology, Hepatology, and Nutrition (ESPGHAN) [21], the target levels of breast milk macronutrients were as follows: 3 g/100 ml of protein, 8.8 g/100 ml of carbohydrates, and 4.4 g/100 ml of fat.

The calculations for target fortification was performed in two steps. First, the macronutrient content of native breast milk was assessed. The additional protein, fat and carbohydrate levels were then calculated on the basis of the ESPGHAN recommendations after subtracting the native breast milk composition in terms of protein, carbohydrates and fat content. The calculated amount of fortifiers was immediately added before the milk was consumed. The following commercially available powdered fortifiers were used: Infant Vita BMF (Dietetic Metabolic Food SRL, Limbiate, Milano, Italy: 48 g protein/100 g, 22 g fat/100 g, 0,47 carbohydrate/100 g); Aptamil BMF (Milupa, Friedrichsdorf, Germany: 25 g protein/100 g, 61 g carbohydrate/100 g); Nidex (Nestlé, Vevey, Switzerland: maltodextrin, 96 g/100 g); Aptamil PS (Milupa, Friedrichsdorf, Germany: 82 g protein/100 g). In the standardized fortification group, the milk had not been analysed; thus, the standard composition of breast milk (protein 1.1 g/100 ml, carbohydrates 7 g/100 ml, and lipids 4.1/100 ml) [7] was assumed. A fixed dose of fortifier was added to the breast milk according to the manufacturer’s instructions. Upon initiation of fortification, the full amount of fortifier was used. The following commercially available powdered fortifiers were used for the standardized fortification group: Aptamil BMF (Milupa, Friedrichsdorf, Germany) and FM85 (Nestlé, Vevey, Switzerland: 20 g protein/100 g, 66 g carbohydrate/100 g). The study period of human milk fortification up until the time of discharge was considered for comparison with the intervention group.

### Data collection and growth assessment

Neonatal characteristics of the intervention group (gestational age, weight, length and head circumference) were prospectively recorded. For the standardized fortification group, the data were calculated retrospectively from the patients’ computerized medical charts.

Gestational age was based on the last menstrual period and first-trimester ultra sonogram. Weight, length, and head circumference were measured according to standard procedures by the same trained nurse [22]. Weight was assessed daily, whereas the length and head circumference were assessed weekly. Infant mass was measured on an electronic scale accurate to the nearest 0.1 g; body length was measured to the nearest 1 mm on a Harpenden neonatometer (Holtain Ltd, UK), and head circumference to the nearest 1 mm using a non-stretch measuring tape. The changes in weight, length and head circumference were calculated weekly.

The daily growth rate was calculated from the start of fortification until discharge and was calculated using an exponential regression model as follows:

$$\left[ { 1000 \, { \times }\;{ \ln }\left( {{\text{W}}_{ 2} /{\text{W}}_{ 1} } \right)} \right]\text{/}\left( {{\text{D}}_{ 2 } - {\text{D}}_{ 1} } \right)$$

where W = weight in grams; D = day; 1 = beginning of the time interval and 2 = end of the time interval [23].

### Osmolality, metabolic assessments and gastrointestinal tolerance of target fortification

The osmolality of the unfortified and targeted fortified breast milk was measured using a freezing point osmometer (Osmometer MIR 300-P, E. Mires, Milan, Italy) before milk administration to the infants. Blood parameters (urea nitrogen n.v. 15–38 mg/dl, creatinine n.v. 0.1–0.7 mg/dl, albumin n.v. 3.8–5.4 g/dl, calcium n.v. 8.40–10.20 mg/dl, phosphorus n.v. 2.7–6.7 mg/dl and alkaline phosphates n.v. <300 U/L levels) were monitored weekly.

Clinical features, including signs of feeding intolerance (e.g., gastric residual volume >50 % of the previous feeding volume, abdominal distension, enteral feeding interruption, and emesis episodes) and stool frequency, were evaluated daily for each infant.

### Statistical analysis

Descriptive data were reported as the mean (standard deviation) (95 % confidence interval), and the significance level was set at 0.05. The sample size was estimated using the daily growth rate. The mean growth rate of the infants fed standard fortified breast milk in our unit was estimated as 12 ± 3 g/kg/day. With a sample size equal to ten and assuming an increase of 3 g/kg/day in the daily growth of infants receiving target fortification compared with standard fortification, the targeted fortification would have resulted in a growth rate of 15 g/kg/day (95 % CI 12.9–17.1). For analysis, inter-group differences in the growth parameters and the daily growth velocity were evaluated using the Mann–Whitney U-test. Statistical analysis was performed using the SPSS version 20 software (Inc, Chicago, IL).

## Results

Ten preterm infants (5 females, 5 males) were enrolled in the intervention group. The standardized fortification group comprised ten preterm infants (4 females, 6 males). The basic characteristics of the enrolled infants in the two groups were similar at birth and at time of enrolment (Table 1). No significant difference in the baseline characteristics between groups was detected.

**Table 1:** Baseline characteristics of the two infant groups at birth and at time of enrolment

Birth	Intervention group Mean (SD)	Standardized fortification group Mean (SD)	p value
Gestational age (weeks)	29.1 (1.03)	29.5 (1.18)	0.5
Weight (g)	1223 (195)	1287 (190)	0.8
Length (cm)	37.2 (3.09)	38.5 (2.26)	0.3
Head circumference (cm)	26.7 (2.84)	27.8 (1.83)	0.5
*Enrolment*			
Gestational age (weeks)	31.4 (0.97)	32.2 (0.63)	0.1
Weight (g)	1412 (231)	1557 (173)	0.3
Length (cm)	38.5 (3.4)	40.2 (2.1)	0.3
Head circumference (cm)	27.3 (2.03)	28.2 (1.13)	0.5

**Fig. 2:**
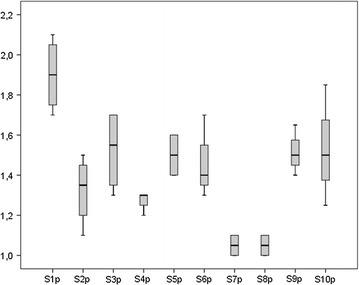
Mean protein (p) content (g/100 ml) of native breast milk of each enrolled subject (S)

**Fig. 3:**
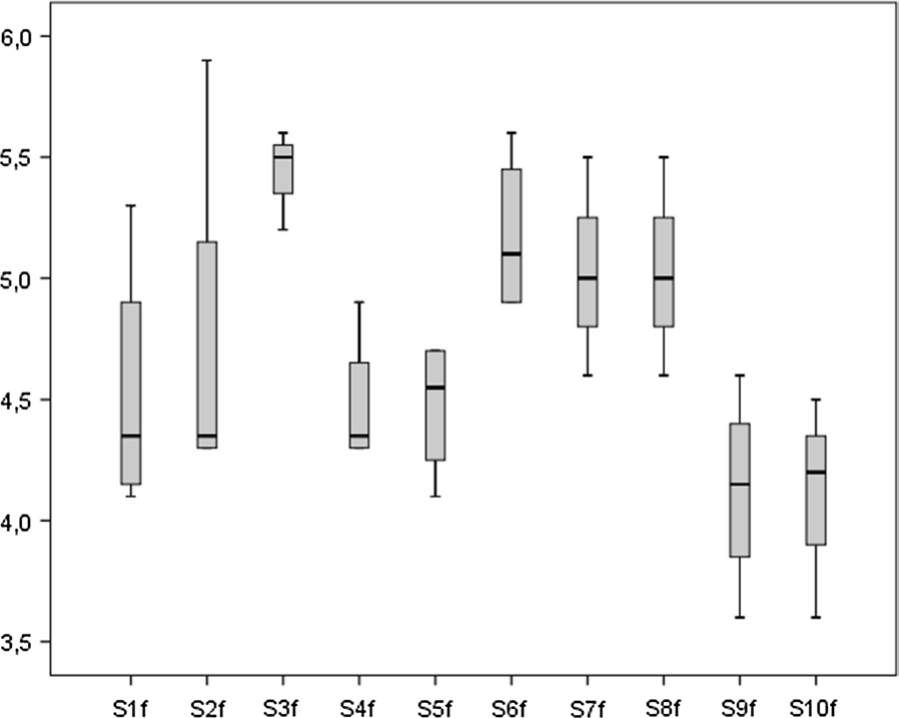
Mean fat (f) content (g/100 ml) of native breast milk of each enrolled subject (S)

**Fig. 4:**
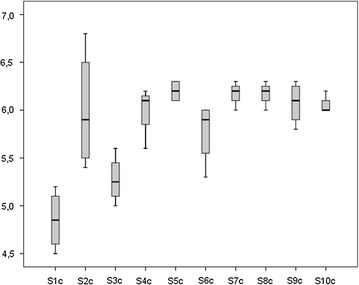
Mean carbohydrate (c) content (g/100 ml) of native breast milk of each enrolled subject (S)

The duration of parenteral nutrition (days) and the day of postnatal life on which enteral feeding was begun were similar in the intervention and standardized fortification groups (20.2 ± 6.7 vs 19.2 ± 5.6, 0.75 ± 0.89 vs 1.1 ± 1.05, respectively). Beginning of fortification (days), full enteral feeding achievement (days) and end of intervention (days) did not differ among groups (16.1 ± 3.0 vs 15.9 ± 3.0, 24.2 ± 7.3 vs 26.8 ± 7.6 and 43.7 ± 7.9 vs 42 ± 9.8, respectively). By the fifth day after fortification, infants in both groups achieved similar feeding volumes (160 ± 6.1 ml/kg/day for intervention group vs 162 ± 11.2 ml/kg/day for standardized fortification group) that were unchanged up until time of discharge. The postmenstrual age (weeks) at time of discharge was similar in the intervention (37 ± 1.6) and the standardized fortification groups (36.8 ± 1.5). The time frame during which human milk was fortified both in the intervention and in the standardized fortification groups was similar and lasted 4–7 weeks. For the intervention group, a total of 118 samples from 24-hour pooled breast milk were analysed. The mean macronutrient content of native breast milk of each enrolled infant is shown in Figs. 2, 3, 4.

**Fig. 5:**
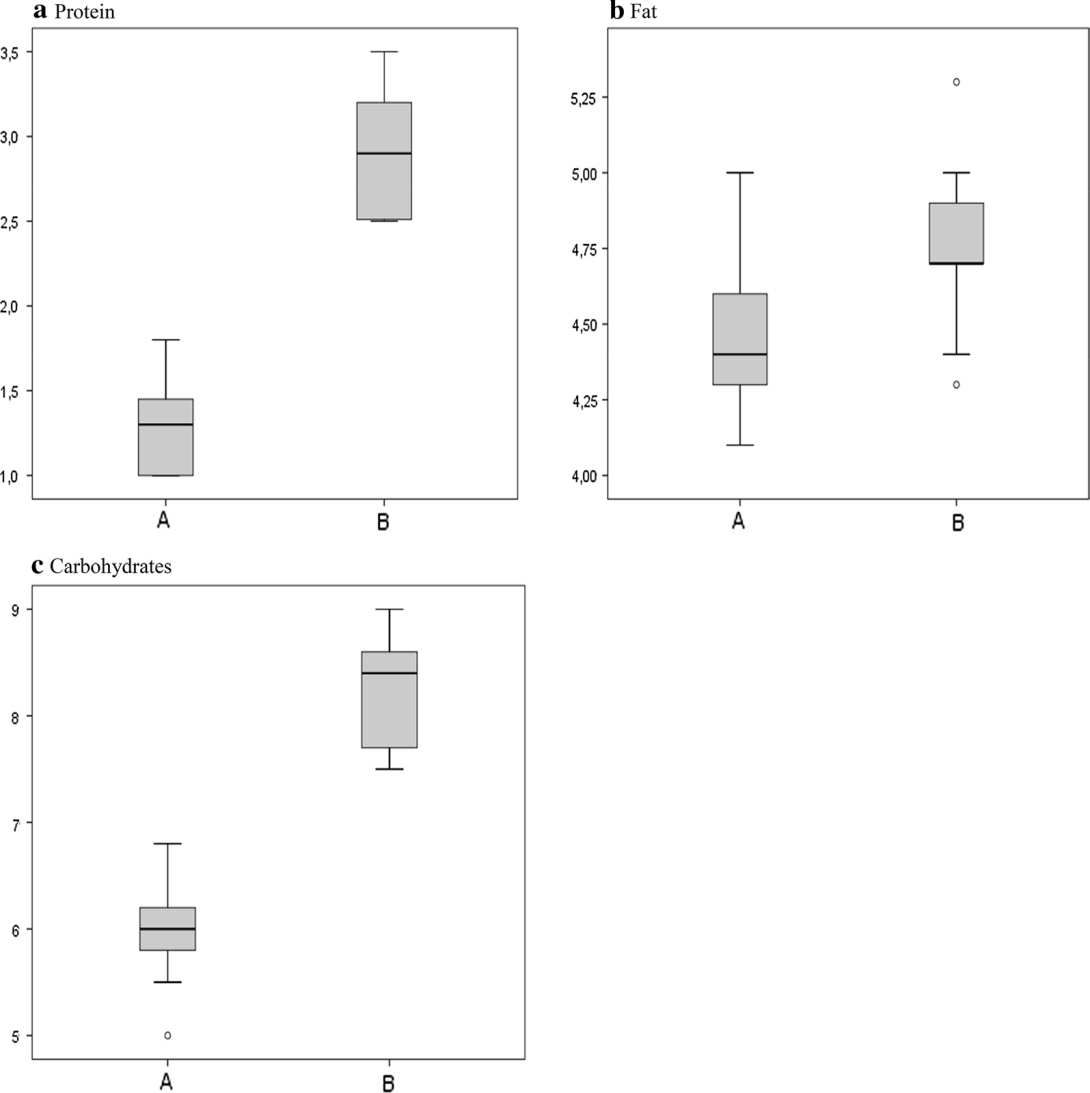
Average protein (**a**), fat (**b**) and carbohydrate (**c**) content (g/100 ml) in native breast milk (A) and targeted breast milk (B)

The variation of macronutrient content in breast milk before and after targeted fortification is shown in Fig. 5. For native breast milk, average protein content ranged from 1.1 to 1.8 g/100 ml, average fat content from 4.1 to 5.0 g/100 ml and average carbohydrate content from 5.0 to 6.8 g/100 ml.

On average, in order to meet ESPGHAN recommendations, 1.4 ± 0.1 g of protein, 2.3 ± 0.5 g of carbohydrate and 0.3 ± 0.1 g of fat per 100 ml were added.

Preterm infants fed targeted fortified breast milk showed better growth compared with preterm infants who received standard fortified breast milk. Specifically, the weekly increase in weight, length, head circumference and daily growth rate were higher in the infants in the intervention group. The weight and length at discharge tended to be higher in the intervention group than in the standardized fortification group, whereas the length of stay and head circumference at time of discharge were similar (Table 2).

**Table 2:** Outcomes of the intervention and standardized fortification groups

	Intervention group Mean (95 % CI)	Historical group Mean (95 % CI)	p value
Weekly weight increase (grams)	205.5 (177–233)	155 (132–178)	0.025*
Weekly length increase (cm)	1.6 (1.1–2.2)	1.1 (0.8–1.5)	0.003*
Weekly head circumference increase (cm)	1.0 (0.9–1.1)	0.9 (0.8–1.0)	0.03*
Daily growth velocity (g/kg/day)	15.7 (14.5–16.9)	12.3 (10.7–13.9)	0.005*
Length of stay (days)	51 (42.9–59.1)	45.5 (40.4–50.6)	0.475
Weight at discharge (grams)	2404.3 (2157.9–2650.7)	2085.5 (1911.8–2259.2)	0.07
Length at discharge (cm)	45.9 (44.2–47.6)	44 (43.1–44.9)	0.07
Head circumference at discharge (cm)	32.2 (31.4–33.1)	31.4 (30.6–32.2)	0.161

* Statistically significant

The infants enrolled in the intervention group showed an adequate metabolic tolerance (urea nitrogen 18 ± 1.2 mg/dl, creatinine 0.3 ± 0.2 mg/dl, albumin 4.2 ± 0.6 mg/dl, calcium 9.40 ± 0.2 mg/dl, phosphorus 3.2 ± 1.8 mg/dl and alkaline phosphates 258 ± 39 U/L levels). In addition, no signs of gastrointestinal intolerance were observed in the infants in the intervention group. The osmolality values (mOsm/kg) of the breast milk were 300 ± 16 before fortification and 376 ± 66 after fortification.

## Discussion

Our preliminary findings suggest that individualized targeted fortification leads to better growth in preterm infants compared with standard fortification without impairing gastrointestinal and metabolic tolerance. On the basis of human milk analysis, the combined addition of single and multicomponent fortifiers allowed the infants in the intervention group to receive the recommended level of each macronutrient according to ESPGHAN guidelines. Targeted fortification considers the variability of macronutrients of native breast milk and, as a result, limits the risk of macronutrient under-intake [13]. Under-intake may have occurred in the standardized fortification group when using standard fortification, resulting in inadequate growth. Human milk protein content after standard fortification may fail to meet the recommended intake for preterm infants [24]. Similarly, probably due to the large variation in the nutritional value of human milk, poor growth rates have been reported in infants fed standardly fortified human milk [13, 25].

Although the infants in the intervention group showed a higher growth rate compared with infants in the standardized fortification group (with a tendency to achieve higher weight and length at time of discharge), the length of hospital stay was similar between the groups. This result is not surprising if we consider that time of discharge is influenced by other clinical conditions, such as the stability of vital parameters, the achievement of independent oral feeding and adequate coordinated sucking abilities.

In recent years, data regarding the benefits of targeted fortification on preterm infant growth have been reported by other authors. Reali et al. [26] showed that the use of targeted fortification led to improved growth rates in a cohort of extremely low birth weight infants (i.e., gestational age ≤30 weeks) in the absence of adverse events. The authors specifically reported a mean daily growth rate of 16.04 g/kg/day, which is higher than the mean weight increase observed in the present study. However, when considering the growth rate of infants with a gestational age comparable with that of the infants enrolled in the present study, the adjusted rate was 14.4 ± 0.75 g/kg/day. Rochow et al. [14] sought to compare the growth pattern of 10 VLBW infants (i.e., gestational age <32 weeks) who were fed breast milk fortified with targeted fortification for at least 3 weeks with a group of infants fed with breast milk fortified according to standard procedure, but they did not observe any significant differences in the daily growth rate between the two groups (19.9 ± 2.7 vs 19.7 ± 3.3 g/kg/day). The authors speculate that as the feeding volumes of infants fed with standard fortification were significantly higher than those of infants in the targeted fortification group, greater benefits regarding weight gain might have been observed if similar feeding volumes were provided in the targeted fortification group. McLeod et al. [27] compared the effect of targeted fortification in a group of preterm infants on growth velocity in comparison to standard fortification and did not find any difference among groups (11.4 ± 1.4 vs 12.1 ± 1.6 g/kg/day). Of note, growth velocity in both groups was lower than recommended (15 g/kg/day) [1].

One strength of our study is that the effect of the targeted fortification was assessed throughout the entire hospital stay, allowing us to gain further insight into the safety and prolonged benefits of targeted fortification on the growth of VLBW preterm infants. The study is limited by the relatively small number of enrolled infants, although it should be considered that the treatment period is relatively long (4–7 weeks, mean 5.6 ± 0.9 weeks). The limited number of subjects is partly due to the strict inclusion criteria, which aimed to exclude the conditions that could negatively interfere with growth. Furthermore, in our NICU, despite the recognized benefits of breast milk on several critical neonatal outcomes, the percentage of infants exclusively fed breast milk is relatively low, in agreement with data from several Italian NICUs [28]. Because this was a single centre study, the findings cannot be generalized; however, it should be noted that the findings were not influenced by any inconsistency of NICU nutritional management.

## Conclusions

These results indicate the feasible implementation of targeted fortification in clinical neonatal practice, although targeted fortification implies an extra workload for health care professionals.

However, this fortification strategy promotes adequate preterm infants weight gain during hospital stay. Considering the beneficial effects of limiting the postnatal growth retardation on later neurodevelopmental and health outcomes, implementation of targeted fortification of breast milk could contribute to the reduction of the burden of long-term adverse outcomes associated with prematurity.

Further randomized controlled studies are necessary to confirm our results and better explore the efficacy and safety of targeted fortification on long-term growth, with particular focus on infants at increased risk for retarded postnatal growth, such as infants with the lowest birth weights and/or affected by comorbidities.

## Abbreviations

VLBW: very low birth weight
NICU: neonatal intensive care unit

## References

[1] American Academy of Pediatrics Committee on Nutrition. Nutritional needs of low-birth-weight infants. Pediatrics.1985;76:976**–**86. 3921937

[2] Horbar JD, Ehrenkranz RA, Badger GJ, Edwards EM, Morrow KA, Soll RF (2015). Weight growth velocity and postnatal growth failure in infants 501 to 1500 grams: 2000–2013. Pediatrics.

[3] Ehrenkranz RA, Dusick AM, Vohr BR, Wright LL, Wrage LA, Poole WK (2006). Growth in the neonatal intensive care unit influences neurodevelopmental and growth outcomes of extremely low birth weight infants. Pediatrics.

[4] American Academy of Pediatrics Section on Breastfeeding: Breastfeeding and the use of human milk. Pediatrics. 2012;129:e827–41. 10.1542/peds.2011-3552 22371471

[5] Human Simmer K, Fortification Milk (2015). Nestlé Nutrition Institute Workshop Ser..

[6] Bauer J, Gerss J (2011). Longitudinal analysis of macronutrients and minerals in human milk produced by mothers of preterm infants. Clin Nutr..

[7] Weber A, Loui A, Jochum F, Bührer C, Obladen M (2001). Breast milk from mothers of very low birthweight infants: variability in fat and protein content. Acta Paediatr.

[8] Rochow N, Jochum F, Redlich A, Korinekova Z, Linnemann K, Weitmann K (2010). Fortification of breast milk in VLBW infants: metabolic acidosis is linked to the composition of fortifiers and alters weight gain and bone mineralization. Clin Nutr..

[9] Pearson F, Johnson MJ, Leaf AA (2013). Milk osmolality: does it matter?. Arch Dis Child Fetal Neonatal..

[10] Kreissl A, Zwiauer V, Repa A, Binder C, Haninger N, Jilma B (2013). Effect of fortifiers and additional protein on the osmolarity of human milk: is it still safe for the premature infant?. J Pediatr Gastroenterol Nutr.

[11] Rochow N, Landau-Crangle E, Fusch C (2015). Challenges in breast milk fortification for preterm infants. Curr Opin Clin Nutr Metab Care..

[12] Arslanoglu S, Moro GE, Ziegler EE (2006). Adjustable fortification of human milk fed to preterm infants: does it make a difference?. J Perinatol.

[13] De Halleux V, Rigo J (2013). Variability in human milk composition: benefit of individualized fortification in very-low-birth-weight infants. Am J Clin Nutr.

[14] Rochow N, Fusch G, Choi A, Chessell L, Elliott L, McDonald K (2013). Target fortification of breast milk with fat, protein, and carbohydrates for preterm infants. J Pediatr.

[15] Reali A, Greco F, Fanaro S, Atzei A, Puddu M, Moi M (2010). Fortification of maternal milk for very low birth weight (VLBW) pre-term neonates. Early Hum Dev..

[16] Fenton TR, Kim JH (2013). A systematic review and meta-analysis to revise the Fenton growth chart for preterm infants. BMC Pediatr..

[17] Bancalari E, Claure N (2006). Definitions and diagnostic criteria for bronchopulmonary dysplasia. Semin Perinatol.

[18] Roggero P, Giannì ML, Orsi A, Amato O, Piemontese P, Liotto N (2012). Implementation of nutritional strategies decreases postnatal growth restriction in preterm infants. PLoS One.

[19] Casadio YS, Williams TM, Lai CT, Olsson SE, Hepworth AR, Hartmann PE (2010). Evaluation of a mid-infrared analyzer for the determination of the macronutrient composition of human milk. J Hum Lact..

[20] Rochow N, Fusch G, Zapanta B, Ali A, Barui S, Fusch C (2015). Target fortification of breast milk: how often should milk analysis be done?. Nutrients..

[21] Agostoni C, Buonocore G, Carnielli VP, De Curtis M, Darmaun D, Decsi T (2010). ESPGHAN Committee on Nutrition. Enteral Nutrient Supply for Preterm Infants: commentary From the European Society for Paediatric Gastroenterology, Hepatology, and Nutrition Committee on Nutrition. J Pediatr Gastroenterol Nutr.

[22] Agostoni C, Grandi F, Giannì ML, Silano M, Torcoletti M, Giovannini M (1999). Growth patterns of breast fed and formula fed infants in the first 12 months of life: an Italian study. Arch Dis Child.

[23] Patel AL, Engstrom JL, Meier PP, Jegier BJ, Kimura RE (2009). Calculating postnatal growth velocity in very low birth weight (VLBW) premature infants. J Perinatol.

[24] Corvaglia L, Aceti A, Paoletti V, Mariani E, Patrono D, Ancora G (2010). Standard fortification of preterm human milk fails to meet recommended protein intake: bedside evaluation by near-infrared-reflectance-analysis. Early Hum Dev..

[25] Henriksen C, Westerberg AC, Rønnestad A, Nakstad B, Veierød MB, Drevon CA (2009). Growth and nutrient intake among very-low-birth-weight infants fed fortified human milk during hospitalisation. Br J Nutr.

[26] Reali A, Greco F, Marongiu G, Deidda F, Atzeni S, Campus R (2015). Individualized fortification of breast milk in 41 extremely low birth weight (ELBW) preterm infants. Clin Chim Acta.

[27] McLeod G, Sherriff J, Hartmann PE, Nathan E, Geddes D, Simmer K (2016). Comparing different methods of human breast milk fortification using measured v. assumed macronutrient composition to target reference growth: a randomised controlled trial. Br J Nutr.

[28] Davanzo R, Monasta L, Ronfani L, Brovedani P, Demarini S (2013). Breastfeeding in Neonatal Intensive Care Unit Study Group. Breastfeeding at NICU discharge: a multicenter Italian study. J Hum Lact..

